# Parent and Provider Perspectives on the Imprecise Label of “Human Milk Fortifier” in the NICU

**DOI:** 10.3390/nu12030720

**Published:** 2020-03-09

**Authors:** Jennifer Canvasser, Amy B. Hair, Jae H. Kim, Sarah N. Taylor

**Affiliations:** 1 NEC Society, PO Box 72271, Davis, CA 95617, USA; 2 Baylor College of Medicine, Texas Children’s Hospital, Houston, TX 77030, USA; abhair@texaschildrens.org; 3 Cincinnati Children’s Hospital Medical Center, University of Cincinnati College of Medicine, Cincinnati, OH 45229, USA; jae.kim@cchmc.org; 4 Yale School of Medicine, New Haven, CT 06520, USA; sarah.n.taylor@yale.edu

**Keywords:** necrotizing enterocolitis, human milk fortifier, patient empowerment, breast milk, neonatal nutrition, prematurity, communication, product labeling, donor milk, NICU parent

## Abstract

In the critical care of preterm infants, feeding is complex and potentially harmful to an immature gastrointestinal system. Parents have expressed the desire to be fully informed about what is being fed to their child, as this places them in the best position to nurture their child’s health. In the parent-engaged setting of the Necrotizing Enterocolitis Symposium, NICU parents expressed concern and confusion about how cow’s milk product and donor human milk product both carry the label “Human Milk Fortifier” (HMF). Accordingly, two online surveys were developed to characterize how the label HMF is used and interpreted in the NICU by parents and providers. Of 774 United States participants, only 21.9% of providers reported consistently describing the source of HMF to parents, and only 20.6% of parents whose child received an HMF product report knowing the source. Parents expressed that they were “not given information” regarding HMF, while both parents and healthcare providers expressed that “the label (HMF) is misleading”. This study documents the ambiguity around the label HMF as well as the need for more specific language and clearer communication.

## 1. Introduction

For new parents, feeding their baby is one of the most primal and meaningful experiences of their lives. When a baby is born prematurely or with a medical condition requiring intensive care, this basic responsibility is taken from new parents and entrusted to professionals. Parents are forced to quickly adapt to parenting in the intensive care setting, where many of their original plans are derailed and they are met with a new and often unexpected reality.

In the critical care of preterm infants, feeding is complex and potentially harmful to an immature gastrointestinal system. Parents are informed of many neonatal intensive care unit (NICU) nutrition and feeding practices, some of which actively rely on maternal production of milk for the infant. For example, parents commonly receive education regarding the lifesaving potential of mother’s own milk and pasteurized human donor milk for preterm and medically fragile infants, which can help protect against necrotizing enterocolitis (NEC), sepsis, and death [1,2,3]. Strategies to optimize the production of mother’s own milk, as well as the rationale for parental consent if pasteurized donor milk is necessary, are often prioritized in parental education.

Human milk alone typically cannot meet the nutritional needs of infants born before 34 weeks’ postmenstrual age (PMA) [4,5]. Accordingly, most NICUs in the United States provide these infants with a Human Milk Fortifier (HMF) that aims to increase the nutritional and caloric density of a mother’s own milk or pasteurized human donor milk. There are two main types of HMF available for use in NICUs based in the United States: one is derived from cow’s milk and the other is derived from pasteurized donor human milk. The term, HMF, does not reflect which type of fortifier is being used. During the NEC Symposium at the University of Michigan (2–5 June 2019, Ann Arbor, MI, USA), the patient-family advocates in attendance described the label HMF as ambiguous, vague, and misleading. These patient-family advocates, whose babies received intensive care, expressed that they felt uninformed when they learned that their babies’ breast milk was being fortified with an HMF product that they misunderstood. These comments led to the development of this survey study examining parental and healthcare provider perceptions of HMF, as well as their understanding of the constituents and purpose of HMF.

## 2. Materials and Methods

### 2.1. Survey Development

This study was reviewed by the Yale University Institutional Regulatory Board (IRB) (New Haven, CT, USA) and was determined to be exempt from IRB approval. Two online surveys were developed for (1) parents and (2) providers in order to characterize how the label “Human Milk Fortifier” (Supplementary Materials) is used and interpreted in the NICU, as well as to establish potential alternative label choices. Each survey contained an introductory paragraph that described the purpose, the eligibility criteria, and how participation in the survey was voluntary. The respondent’s completion of the survey served as documentation of consent to participate in the study.

The survey questions included both multiple choice and open-ended text fields, which were developed by parents whose children have been affected by NEC, in partnership with neonatologists affiliated with the NEC Society (www.necsociety.org). The survey questions stemmed from the lived experience of parents in the NICU. NICU parents selected 19 adjectives from a list of some of the most common human feelings which could be used to describe potential emotions associated with various infant feeding types. Prior to dissemination, both surveys were distributed to an expert panel of parents and scientists from the NEC Society for face validity and question optimization.

The first survey was developed for NICU parents > 18 years old from the United States. These questions were adapted to address parents whose infant(s) received care in an NICU, including those parents whose infant(s) have been affected by NEC or have died. Optional demographic information included parent age, education level, and race/ethnicity categories.

The second survey was directed to healthcare providers in the NICU, including nurses, physicians, nurse practitioners, physician assistants, lactation consultants, dietitians, and other members of the clinical care team in the NICU. Providers had the option to include years of experience and race/ethnicity categories.

### 2.2. Survey Distribution

The two surveys were created and distributed through the online-based survey platform Qualtrics© (Seattle, Washington, USA). Links to the survey were disseminated via the NEC Society’s listserv, social media pages, and blog. The NICU Parent Network also circulated links to the survey on their listserv and social media pages. The provider survey was emailed to contacts at 11 United States level III/IV academic NICU centers who were requested to distribute the survey to all NICU clinical staff. The surveys were available to respondents from 15 August 2019, through to 23 September 2019.

### 2.3. Analysis

These surveys were exploratory and, therefore, no sample size calculations were performed. Survey data was compiled into summary statistics including the proportion of respondents providing each answer. Test answers were coded for themes using Dedoose Version 7.0.23, web application for managing, analyzing, and presenting qualitative and mixed method research data (2016). Los Angeles, California, USA: SocioCultural Research Consultants, LLC. Relative risk (RR) with 95% confidence intervals (CI) were calculated by Chi-squared analysis.

## 3. Results

### 3.1. Parent Survey

#### 3.1.1. Demographics

The parent survey had 395 respondents in the United States. Demographics are presented in Table 1. The parent respondents who opted to answer the demographic questions were primarily white, college-educated, 30–39 years of age, with a child born between 2011 and 2019 who received NICU care.

#### 3.1.2. Feeding Type

Among the 395 parent respondents, 90.6% reported that their child received their mother’s own milk in the NICU, 71.1% reported that their child received HMF, and 44.5% remember being told that human milk helps to reduce the risk of NEC.

Regarding formula feeding, 48.6% of parents reported that their child received formula in the NICU, and of those 192 parent respondents, 68% reported that they were asked to give consent prior to their child receiving formula.

#### 3.1.3. Necrotizing Enterocolitis

In this survey, 49.9% of parent respondents reported that their child was diagnosed with NEC in the NICU. Of these, 30.5% had a child who died, with 96.7% of these cases due to NEC or complications related to NEC.

#### 3.1.4. Adjectives Describing Mother’s Own Milk or Fortification

**Figure 1:**
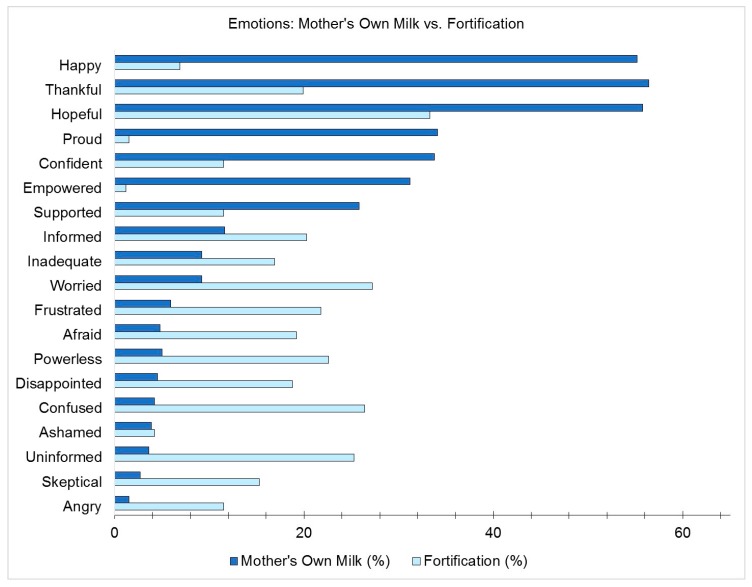
The percentage of respondents choosing each listed adjective in response to the questions “How did you feel about your baby receiving mother’s milk?” and “Reflecting back to your time in the NICU with your baby, how did you feel about fortification then?”.

Parents were asked to choose up to five adjectives in response to the questions “How did you feel about your baby receiving mother’s milk?” and “Reflecting back to your time in the NICU with your baby, how did you feel about fortification then?” The percentage of parent respondents who chose each adjective for each question is shown in Figure 1.

### 3.2. Provider Survey

#### Demographics

The provider survey had 379 respondents in the United States. Provider demographics are provided in Table 2. Provider respondents were primarily white nurses and physicians who have provided NICU care for over 10 years.

### 3.3. Human Milk Fortifier Information and Labeling

Of the 281 parent respondents who answered that their child received HMF, 60.1% reported that they were told about HMF prior to their child receiving it and 82.6% of those parents reported being told why their child was receiving HMF. However, only 20.6% report knowing the source of their child’s HMF. Reflecting back to their time in the NICU, only 8.8% of parent respondents interpreted the product as potentially meaning a cow’s milk-based product. In fact, many parents (n = 48%) did not know that HMF could be derived from cow’s milk or pasteurized human donor milk until they participated in this survey study: provider report of communication with parents regarding HMF.

Of the 379 provider respondents, 21.9% answered that they consistently inform parents as to whether their child’s HMF is cow’s milk- or human milk-based, 23.3% answered that they sometimes inform parents, and 54.8% stated that they do not inform parents of this information.

Parent and provider responses as to what the term “HMF” means to them are shown in Table 3, and recommendations to improve clarity are provided in Table 4. Regarding fortifiers derived from cow’s milk, 73% of parent respondents and 54% of provider respondents thought that bovine fortifiers should include the label “Cow’s Milk” to improve clarity.

In an analysis of provider characteristics and their relationship with providing parental information regarding fortifier sources, providers with greater experience (at least 10 years) were not significantly more likely to inform parents when compared to providers with less experience (RR 1.01, 95% CI 0.86–1.18). However, when comparing provider role in the NICU, nurses were less likely to inform parents about the source of HMF compared to other providers (RR 1.63, 95% CI 1.35–1.96).

#### Qualitative Survey Data

Throughout the open-ended text field in the parent survey, phrases including “the label is misleading” and “it should be clear” were mentioned in the context of HMF labeling. The most common theme expressed by parents was that they were “not given information”, with 21 parent respondents providing open-ended text that included this theme. Excerpts from parent comments that best represent these themes include:

*“I wish I would have been told that the fortifier was cow’s-milk based. The name is way too confusing.”*

*“It should be very clear whether the fortifier is derived from cow’s-milk formula or breast milk. A label with “human milk” should only mean it’s FROM humans.”*

*“While in the NICU with my baby, I had to ask what HMF stood for and I only really understood what it was once we were home and I could see the bottle and read the ingredient label.”*

*“Until today, I had no understanding of what “fortifier” meant when nurses used that term. It’s disappointing how uninformed parents are in the NICU during such a vulnerable time.”*

The theme of “the label is misleading” was mentioned by providers in the open-ended text field, as shown by the following example:

*“The labeling of our cow’s milk-based fortifiers is misleading because it still has the label “human milk.”*

## 4. Discussion

To our knowledge, this is the first study to formally document how the label HMF is used and interpreted by parents and providers in the NICU. In this survey study, we found that while most parents (n = 82.6%) were told why their child was receiving HMF, few parents (n = 20.6%) reported knowing the source of their child’s HMF. Reflecting back to their time in the NICU, only 8.8% of parent respondents interpreted the product as potentially meaning a cow’s milk-based product. In fact, until they participated in this survey study, many parents (n = 48%) did not know that HMF could be derived from cow’s milk or pasteurized human donor milk. Only 21.9% of providers reported consistently describing the source of HMF to parents. The lack of a transfer of information from provider to parent was also evident in the qualitative open-ended text responses, where parents expressed a feeling of being misinformed and needing clarity, with the most common theme being “not informed”.

Most providers acknowledged that HMF can be derived from cow’s milk or human milk, and yet provider awareness did not translate into parental knowledge in this study. While nurses were significantly less likely to inform parents of the derivation of their child’s HMF, the study results cannot determine the basis for this lack of transfer of information. This study raises the possibility that providers have this information but do not find it pertinent to share it with parents. It is also possible that some providers may find it challenging to clearly articulate the differences between fortifiers to parents, or perhaps they do not characterize their professional role as having to inform parents of this type of information. Nevertheless, parents rely on the bedside team as a vital source of information. Clearly, there are a plethora of issues for NICU providers to determine if, how, and when to share with NICU parents. Providers may fear that sharing more information with parents will only further overwhelm them. On the contrary, NICU parents are empowered when information is presented with empathy as early as possible [6]. Gadepalli and colleagues found that informed parents are more satisfied with care, even when their infant’s outcome is poor, because it allows them to better engage and contribute as a member of their child’s care team. Indeed, there are opportunities for education and further research focused on communication between NICU providers and parents, which go beyond the labeling of HMF products.

Given that both NICU parents and providers find the label HMF to be ambiguous and misleading, there is an urgent need for an alternative, more precise product name. This study demonstrates that the label HMF thwarts an NICU parent’s ability to be fully informed and a provider’s ability to deliver clear information to families. Accordingly, it is vital for providers to not only fully understand the products that they are prescribing to their patients, but also to have the capacity to clearly articulate to NICU parents the basic components and purpose of the products they are prescribing.

Clarity in labeling is not only a priority for parents and providers, but also for the U.S. Food and Drug Administration. Under Section 403 (a) (1) of the Federal Food, Drug, and Cosmetic Act (FD&C Act) (21 U.S.C. 343 (a) (1)), food labeling, including that concerning infant formula and human milk fortifiers, must be truthful and not misleading. This guidance specifically addresses function claims such as the benefit of the formula/fortifier, but significant emphasis is placed on removing any “misleading” information. The results of this study support the argument that the current label HMF is misleading.

Gooding and colleagues emphasized the importance of open and honest communication between parents and providers in the NICU as it fosters family-centered care (FCC). FCC takes a holistic approach to optimizing infant outcomes by actively engaging the parents. In FCC, providers recognize their role is to care for patients in the context of their family and community. Accordingly, providers engage parents in decision-making and serve as a mentor as parents learn how to care for their medically fragile infant. Importantly, FCC can help to improve parental mental health, increase parent–infant bonding, decrease the NICU length of stay, and improve developmental outcomes [7]. Given the gravity of clear communication between NICU parents and providers, a more transparent product label for HMF should be of high priority.

Communication between parents and providers may potentially affect how an NICU parent engages in their child’s care. Pineda et al. stressed that when NICU parents tune in and provide hands-on care for their baby, they actually help to optimize their infant’s brain development and long-term outcomes. Nurturing interactions between the parent and infant in the NICU may also strengthen parent–child attachment, which can further support a child’s long-term healthy development [8]. When providers support parents in understanding what is being fed to their child, they may help to foster the parents’ engagement and active participation. NICU parents who are empowered with information may be better able to contribute and engage in a meaningful way. Parents seek to know precisely what is being fed to their baby, which can help them serve as informed and engaged members of their child’s care team. Yet, the label HMF is imprecise.

This study has multiple limitations that warrant mention. Convenience sampling was used, as a random sample of NICU parents and providers was not available. Since the NEC Society developed the surveys, it is not surprising that 49.9% of parent respondents had a child that was affected by NEC. Additionally, the self-selected parents and providers who participated may bias the sample towards individuals who have stronger perspectives about the label HMF. Parents and providers may also have recall bias due to the retrospective nature of the study. Furthermore, parents and providers of diverse backgrounds were underrepresented.

## 5. Conclusions

This study demonstrates the need to clarify the term and concept of HMF” to parents and specify the source of the product. It may b/e beneficial to explore formal label changes to these commercial products.

## Figures and Tables

**Table 1:** Parent demographics.

	Parent Cohort % (n)
**Age (years):**	**N = 345**
**Under 18**	0.5% (2)
**18–29**	20% (68)
**30–39**	57% (195)
**40–50**	21% (74)
**50 Years or above**	1.5% (6)
**Race/Ethnicity:**	**N = 358**
**White**	85% (305)
**Hispanic or Latino**	7% (24)
**Black**	3% (11)
**Asian/Asian Indian**	3% (12)
**Other**	2% (6)
**Formal Education:**	**N = 344**
**High School Degree**	20% (69)
**College Graduate**	45% (156)
**Post-Graduate Degree**	30% (101)
**Other**	5% (18)
**Year Child was Born:**	**N = 395**
**2010 and Below**	14% (54)
**2011–2013**	16% (62)
**2014–2016**	25% (101)
**2017–2019**	45% (177)
**Child Gestational Age at Birth:**	**N = 395**
**22–24 weeks**	17% (65)
**25–28 weeks**	35% (137)
**29–33 weeks**	29% (115)
**34–36 weeks**	12% (49)
**37–42 weeks**	7% (29)

**Table 2:** Provider demographics.

	Provider Cohort
	N = 379
	% (n)
**Position:**	
**Nurse**	52% (196)
**Physician**	28% (108)
**Neonatal Nurse Practitioner**	8% (29)
**Registered Dietitian**	6% (21)
**Fellow/Resident**	4% (15)
**Physician Assistant**	1% (5)
**Lactation Consultant**	1% (5)
**Length of Providing NICU Care:**	
**More Than 20 Years**	27% (101)
**10–19 years**	31% (117)
**5–9 years**	25% (96)
**1–4 years**	15% (58)
**Less Than a Year**	2% (7)
**Race/Ethnicity:**	
**White**	80% (305)
**Asian/Asian Indian**	8.5% (32)
**Hispanic or Latino**	5% (18)
**Black**	3% (11)
**Other**	3.5% (13)

**Table 3:** Parent and provider responses regarding the term “human milk fortifier”.

%(n) Choosing the Response *	Parent Survey:“In the NICU, What Did HMF Mean to You?”	Parent Survey:“Today, What Words Describe HMF?”	%(n) Choosing the Response *	Provider Survey:“What Does the Label HMF Mean to You?”
**Cow’s milk-based product**	10% (35)	22% (75)	**Cow’s milk-based product**	52% (194)
**Human milk-based product**	21% (71)	49% (171)	**Human milk-based product**	35% (133)
**Vitamins and minerals**	27% (93)	19% (67)	**Concentrated formula**	12% (45)
**Additional calorie source**	64% (221)	44% (151)	**Supplement in addition to human milk**	39% (146)
**Not sure**	13% (44)	14% (48)	**Additive to human milk**	83% (313)
**Other**	7% (24)	4% (13)	**Other**	2% (8)

* Able to choose more than one response.

**Table 4:** Parent and provider recommendations for the best way to describe Human Milk Fortifier (HMF).

Response	Parent (n = 344)	Provider (n = 377)
**For cow’s milk-based fortifier**		
**Cow’s milk-based fortifier**	73% (251)	54% (203)
**Concentrated formula**	20% (70)	8% (31)
**Human milk fortifier**	4% (13)	27% (100)
Other*	3% (10)	11% (43)
**For human milk-based fortifier**		
**Human milk-based fortifier**	53% (183)	59% (224)
**Donor milk fortifier**	24% (83)	19% (70)
**Human milk fortifier**	20% (67)	17% (66)
Other*	3% (10)	5% (19)

* Other suggestions for “cow’s milk-based fortifier” from parents included “bovine milk fortifier”, “non-human milk fortifier”, and “fortifier”. Other suggestions for “cow’s milk-based fortifier” from providers included “cow’s milk-based human milk fortifier”, “nutrient-enriched fortifier for human milk”, “fortifier that contains broken-down cow’s milk proteins as well as many other nutrients”, “bovine-based”, “cow’s milk-based fortifier for human milk”, “bovine-derived fortifier”, “formula-based fortifier”, “cow’s milk-derived fortifier”, “dairy-based fortifier”, “supplement for growth”, “milk fortifier”, “an additive to your breast milk that comes from cow’s milk”, “cow’s milk-based human milk fortifier”, “fortifier for your milk that is cow’s milk-based”, “fortifier to human milk containing hydrolyzed cow’s milk protein”, and “fortifier”. Other suggestions for “human milk-based fortifier” from parents included “donor breast milk”, “donor milk-based fortifier”, “breast milk-based fortifier”, and “human donor milk fortifier”. Other suggestions for “human milk-based fortifier” from providers included “donor human milk-based fortifier”, “donor human milk-based fortifier for human milk”, “purchase-pooled human milk-based fortifier”, “human milk-derived fortifier”, “human milk-based human milk fortifier”, “human milk-derived fortifier”, “concentrated human milk”, “donor milk-based fortifier”, “human milk-derived human milk fortifier”, “an additive to your breast milk that comes from donated human milk”, and “fortifier for your milk that is made from pasteurized donor human milk”.

## Supplementary Materials

The Human Milk Fortifier (HMF) label survey questions for parents as well as for providers are available as supplementary materials online at https://www.mdpi.com/2072-6643/12/3/720/s1.
