# Supplementary material for: Parent and Provider Perspectives on the Imprecise Label of “Human Milk Fortifier” in the NICU
Source: Nutrients. 2020 Mar 9;12(3):720. doi: 10.3390/nu12030720 (PMC7146547; doi:10.3390/nu12030720)
Supplement: Supplementary file 1 [file nutrients-12-00720-s001.zip › Supplement HMF survey questions /Supplement_Parent_HMF survey questions.pdf]

Parent experience and understanding of the label "Human Milk Fortifier"

Start of Block: Default Question Block

0 The NEC Society has launched a research project to gain a better understanding of how the label "Human Milk Fortifier" is interpreted by parents in the NICU. If your child spent time in the NICU and/or was diagnosed with necrotizing enterocolitis (NEC), you are encouraged to consider sharing your experience by participating in this short survey.

If you've had more than one baby in the NICU and/or diagnosed with NEC, then please complete a new survey for each child.

Participants must be at least 18 years old. This is a questionnaire-based survey being conducted by Sarah Taylor, Jennifer Canvasser, Amy Hair, and Jae Kim through Yale University. The only activity for this study is completion of this survey. The survey will take approximately 5 minutes to complete. Your responses will be confidential, and we will not collect identifying information such as your name, email address, or IP address. This study will not benefit you but may provide information to improve preterm infant clinical care in the future. You will not be paid for participation. Taking part in this study is your choice. You may choose not to participate. If you decide to participate in this research survey, you may withdraw your participation by stopping the survey at any time. Your completion and submission of this survey serves as your consent to participate in this study. Questions about this study should be addressed to Dr. Sarah Taylor at Yale University at 203-688-2320 or PO Box 208064, New Haven, CT 06520.

If you are at least 18 years old, have or had a child in the NICU and/or diagnosed with NEC, and agree to participate in this study, please start the survey.

Q1 Where do you live?

▼ United States of America ... Zimbabwe

Display This Question:

If List of Countries = United States of America

Q2 Where in the United States do you live?

▼ Alabama ... I do not reside in the United States

3 Did your child spend time in the neonatal intensive care unit (NICU)?

- ◯ Yes
- ◯ No

Q4 What year was your child born?

- Before 2010
- 2011
- 2012
- 2013
- 2014
- 2015
- 2016
- 2017
- 2018
- 2019

Q5 Your child was born:

- 37 - 42 weeks gestational age
- 34 - 36 weeks gestational age
- 29 - 33 weeks gestational age
- 25 - 28 weeks gestational age
- 22 - 24 weeks gestational age

Q6
Was your baby diagnosed with necrotizing enterocolitis (NEC), a severe condition of bowel inflammation?

- ◯ Yes, my baby was diagnosed with NEC.
- ◯ No, my baby was not diagnosed with NEC.

Display This Question:

If Was your baby diagnosed with necrotizing enterocolitis (NEC), a severe condition of bowel inflamm... = Yes, my baby was diagnosed with NEC.

Q7 Did your baby survive NEC?

- ◯ Yes, my baby survived.
- ◯ No, my baby passed away.

Display This Question:

If Did your baby survive NEC? = No, my baby passed away.

Q8 We are so sorry for your loss. Did your baby pass away from NEC or complications of NEC?

- ◯ Yes, my baby passed away from NEC or complications from NEC
- ◯ No, my baby passed away from something other than NEC

Display This Question:

If Did your child spend time in the neonatal intensive care unit (NICU)? = Yes

Q9 Did your baby receive any mothers milk in the NICU?

- ◯ Yes
- ◯ No

Display This Question:

If Did your baby receive any mothers milk in the NICU? = Yes

Q10 How did you feel about your baby receiving mothers milk? (select up to 5)

- ☐ Afraid
- ☐ Angry
- ☐ Ashamed
- ☐ Confident
- ☐ Confused
- ☐ Disappointed
- ☐ Empowered
- ☐ Frustrated
- ☐ Happy
- ☐ Hopeful
- ☐ Inadequate
- ☐ Informed
- ☐ Powerless
- ☐ Proud
- ☐ Skeptical
- ☐ Supported

☐ Thankful

☐ Uninformed

☐ Worried

Display This Question:

If Did your child spend time in the neonatal intensive care unit (NICU)? = Yes

Q11 DId your baby receive any formula in the NICU?

○ Yes

○ No

○ Only cow's milk-based fortifier that was added to mothers/donor milk

Display This Question:

If DId your baby receive any formula in the NICU? = Yes

Or DId your baby receive any formula in the NICU? = Only cow's milk-based fortifier that was added to mothers/donor milk

Q12 Were you asked to give your consent before formula was given to your baby?

○ Yes

○ No

○ Unsure

Q13 Did your baby receive a Fortifier that was added to mother's own milk or donor milk?

◯ Yes

◯ No

◯ Unsure

Display This Question:

If Did your baby receive a Fortifier that was added to mother's own milk or donor milk? = Yes

Q14 Did you know that your baby was going to receive a Fortifier before it was given?

◯ Yes, I was told about the Fortifier before it was given.

◯ No, I was not told about the Fortifier before it was given.

◯ I am unsure.

Display This Question:

If Did your baby receive a Fortifier that was added to mother's own milk or donor milk? = Yes

Q15 Were you told why the Fortifier would be added to your baby's breast/donor milk?

◯ Yes, I was given a reason(s) for the Fortifier to be added.

◯ No, I was not given a reason(s) for the Fortifier to be added.

◯ I am unsure.

Display This Question:

If Did your baby receive a Fortifier that was added to mother's own milk or donor milk? = Yes

Q16 When the Fortifier was given to your baby in the NICU, were you told whether your baby's Fortifier was cow-based or pasteurized donor human milk-based?

- ◯ No, I was not told.
- ◯ Yes, I was told my baby's Fortifier was cow's milk-based.
- ◯ Yes, I was told my baby's Fortifier was pasteurized donor human milk-based.

Display This Question:

If Did your child spend time in the neonatal intensive care unit (NICU)? = Yes

Q17 During your time in the NICU with your baby, were you told that human milk (either from mother or a donor) can help to reduce the risks of necrotizing enterocolitis?

- ◯ Yes, in the NICU I was told that human milk can help reduce the risks of NEC.
- ◯ No, in the NICU I was not told that human milk can help to reduce the risks of NEC.
- ◯ I am unsure.

Display This Question:

If When the Fortifier was given to your baby in the NICU, were you told whether your baby's Fortifie... = No, I was not told.

Q18 Today, do you know if your baby's Fortifier was from pasteurized donor human-milk or cow's-milk?

- ◯ No, I am not sure.
- ◯ Yes, my baby's Fortifier was from a cow's milk-based product.
- ◯ Yes, my baby's Fortifier was from pasteurized donor human milk.

Display This Question:

If Did your baby receive a Fortifier that was added to mother's own milk or donor milk? = Yes

Q19 Reflecting back to your time in the NICU with your baby, how did you feel about Fortification then? (select up to 5)

- ☐ Afraid
- ☐ Angry
- ☐ Ashamed
- ☐ Confident
- ☐ Confused
- ☐ Disappointed
- ☐ Empowered
- ☐ Frustrated
- ☐ Happy
- ☐ Hopeful
- ☐ Inadequate
- ☐ Informed
- ☐ Powerless
- ☐ Proud
- ☐ Skeptical
- ☐ Supported

- [ ] Thankful
- [ ] Uninformed
- [ ] Worried

Display This Question:

If Did your child spend time in the neonatal intensive care unit (NICU)? = Yes

Q20 Reflecting back to your time in the NICU with your baby, what did "Human Milk Fortifier" mean to you then?

- [ ] Cow’s milk-based product
- [ ] Human-milk based product
- [ ] Vitamins and minerals
- [ ] Additional calorie source
- [ ] Other: ____
- [ ] Unsure

Q21 There are two main Fortifiers used in NICUs. One is a cow's milk-based Fortifier derived from cow's milk-based formula. Another is a human donor milk based Fortifier derived from pasteurized donor breast milk

When did you learn the difference between human-milk based and cow's milk-based Fortifiers?

- ○ When my baby was in the NICU
- ○ After my baby was diagnosed with NEC
- ○ After my baby came home
- ○ After my baby died
- ○ Today

Q22 Today, what words would you use to describe "Human Milk Fortifier?"

- ▢ Cow's mlk-based product
- ▢ Human-milk based product
- ▢ Vitamins and minerals
- ▢ Additional calorie source
- ▢ Other: ____
- ▢ Unsure

Q23 What is the best way to describe a Fortifier made from COW'S-MILK?

- ○ Cow’s milk-based fortifier
- ○ Concentrated formula
- ○ Human Milk Fortifier
- ○ Other: ____

Q24 What is the best way to describe a Fortifier made from PASTEURIZED DONOR HUMAN MILK?

- ○ Human-Milk Based Fortifier
- ○ Donor-Milk Fortifier
- ○ Human Milk Fortifier
- ○ Other: ____

Q25 What is your age? (optional)

- ○ 50 years +
- ○ 40 - 50 years
- ○ 30 - 39 years
- ○ 18 - 29 years
- ○ Under 18 years

Q26 What is your formal education? (optional)

- ○ Some high school
- ○ High school graduate
- ○ College graduate
- ○ Post-graduate degree
- ○ Other ____

Q27 What is your race or ethnicity? (please select all that apply) (your response is optional)

- ☐ Asian/Asian Indian
- ☐ Black/African American
- ☐ Hispanic, Latino, or Spanish
- ☐ Middle Eastern or North African
- ☐ Native American
- ☐ Pacific Islander
- ☐ White
- ☐ Other ____

*

Q28 Would you like to share any other thoughts or feedback with the NEC Society?

____

End of Block: Default Question Block
