# Supplementary material for: Parent and Provider Perspectives on the Imprecise Label of “Human Milk Fortifier” in the NICU
Source: Nutrients. 2020 Mar 9;12(3):720. doi: 10.3390/nu12030720 (PMC7146547; doi:10.3390/nu12030720)
Supplement: Supplementary file 1 [file nutrients-12-00720-s001.zip › Supplement HMF survey questions /Supplement_Provider_HMF survey questions.pdf]

Provider experience and understanding of the label "Human Milk Fortifier"

Start of Block: Default Question Block

Q0 The NEC Society has launched a research project to gain a better understanding of how the label "Human Milk Fortifier" is used and interpreted by providers in the NICU. You are encouraged to share your expertise by participating in this short survey.

Participants must be at least 18 years old. This is a questionnaire-based survey being conducted by Sarah Taylor, Jennifer Canvasser, Amy Hair, and Jae Kim through Yale University. The only activity for this study is completion of the survey. The survey will take approximately 5 minutes to complete. Your responses will be confidential, and we do not collect identifying information such as your name, email address, or IP address. This study will not benefit you but may provide information to improve preterm infant clinical care in the future. You will not be paid for participation. Taking part in this study is your choice. You may choose not to participate. If you decide to participate in this research survey, you may withdraw your participation by stopping the survey at any time. Your completion and submission of this survey serves as your consent to participate in this study. Questions about this study should be addressed to Dr. Sarah Taylor at Yale University at 203-688-2320 or PO Box 208064, New Haven, CT 06520.

If you provide care to premature and medically fragile infants at increased risk of NEC in the NICU, are at least 18 years-old, and agree to participate in this study, please start the survey.

Q1 Where do you live?

▼ United States of America ... Zimbabwe

Display This Question:

If List of Countries = United States of America

Q2 Where in the United States do you live?

▼ Alabama ... I do not reside in the United States

Q3 What best describes you?

- ◯ Nurse (RN, BSN)
- ◯ Nurse Practitioner (NNP)
- ◯ Registered Dietitian (RD)
- ◯ Physician Assistant (PA)
- ◯ Lactation Consultant (LC)
- ◯ Resident
- ◯ Fellow
- ◯ Hospitalist
- ◯ Attending physician

Q4 What does the label "Human Milk Fortifier" mean? (check all that apply)

- ☐ Cow's milk based product
- ☐ Pasteurized human donor milk based product
- ☐ Concentrated formula
- ☐ Supplement in addition to human milk
- ☐ Additive to human milk
- ☐ Other: ____

Q5 There are two main Fortifiers used in NICUs. One is a cow's milk-based product derived from cow's milk-based formula. Another is a human donor milk-based Fortifier derived from pasteurized donor breast milk.

Do you tell parents whether their baby's Fortifier is cow's milk based or pasteurized donor milk based?

- ○ Yes
- ○ No
- ○ Sometimes

Q6 What is the best way to describe a Fortifier made from COW'S MILK?

- ◯ Cow's milk based fortifier
- ◯ Concentrated formula
- ◯ Human Milk Fortifier
- ◯ Other: ________________________________

Q7 What is the best way to describe a Fortifier made from PASTEURIZED DONOR HUMAN MILK?

- ◯ Human-Milk Based Fortifier
- ◯ Donor-Milk Fortifier
- ◯ Human Milk Fortifier
- ◯ Other: ________________________________

Q8 How long have you been providing care to infants in the NICU?

- ◯ More than 20 years
- ◯ 10 - 19 years
- ◯ 5 - 9 years
- ◯ 1 - 4 years
- ◯ Less than a year

Q9 What is your race or ethnicity? (select all that apply) (your response is optional)

- ☐ Asian/Asian Indian
- ☐ Black/African American
- ☐ Hispanic, Latino, or Spanish
- ☐ Middle Eastern or North African
- ☐ Native American
- ☐ Pacific Islander
- ☐ White
- ☐ Other ____

End of Block: Default Question Block
